# Genetic analysis of neurodegeneration.. the end game

**DOI:** 10.1186/1750-1326-8-S1-O12

**Published:** 2013-09-13

**Authors:** John Hardy

**Affiliations:** 1 Reta Lilla Weston Research Laboratories and Department of Molecular Neuroscience, UCL Institute of Neurology, London, WC1N 3BG, UK

## 

In my talk I will discuss how genetic analysis now allows us to dissect both causative and predisposing genes for neurodegenerative diseases. Mendelian diseases can be found by positional cloning and sequencing... rare, high risk variants can be found by exome sequencing and burden analysis and common low risk variants can be found by genome wide association analyses. I will show how, when loci for disease are found, they map functionally into specific biochemical pathway. This property is also helpful in identifying new loci for which the statistical evidence is less than convincing. With regard to Alzheimer’s disease, the well known mendelian genes are APP and PSEN1 and PSEN2. In addition to apoe, genome wide association studies have identified now nearly 20 loci which map onto four pathways, cholesterol metabolism, the innate immune system, endosomal vesicle recycling and protein ubiquitination. At the time of writing this abstract, TREM2, which is involved in the innate immune system and control of miscroglial activation is the only declared rare variant of high effect (o.r. ~5), but several other genes have data in the publication process. I will discuss these issues and update on the current state of play for Alzheimer genetics and describe how we can move towards the completion of genetic analysis.

